# Supplementary material for: In vitro spermatogenesis in isolated seminiferous tubules of immature mice
Source: PLoS One. 2023 Apr 6;18(4):e0283773. doi: 10.1371/journal.pone.0283773 (PMC10079070; doi:10.1371/journal.pone.0283773)
Supplement: S1 File — (PDF) [file pone.0283773.s001.pdf]

S1 Fig. Acr/H3 double-Tg mouse testis as a system for monitoring spermatogenesis.

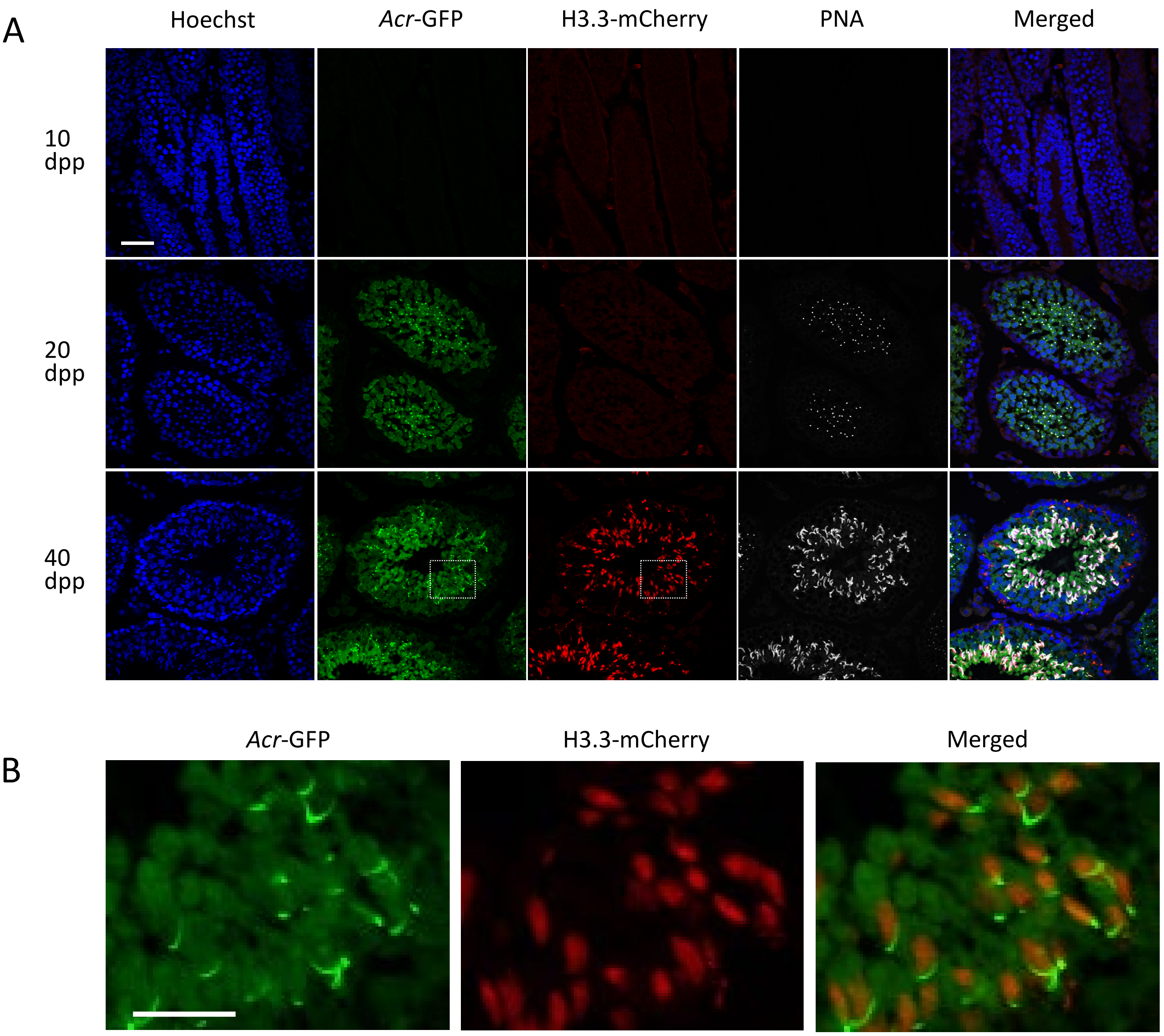

Acr/H3 double-Tg mouse testis as a system for monitoring spermatogenesis.
(A) Testes of Acr/H3 double-Tg mice at ages of 10, 20, and 40 dpp were immunohistochemically examined with antibodies to GFP (green), mCherry (red), and PNA (gray). Nuclear staining was performed with Hoechst (blue). (B) Magnified immunohistochemical view of 40 dpp mouse testis. Dashed rectangular areas in A are enlarged in B. Elongating spermatids were stained with GFP and mCherry in the acrosome and nucleus, respectively. This double-staining pattern can be a reliable signature for identifying elongating spermatids. Scale bars: 50 µm (A), 20 µm (B).

S2 Fig. Seminiferous tubule PDMS ceiling (STPC) chip.

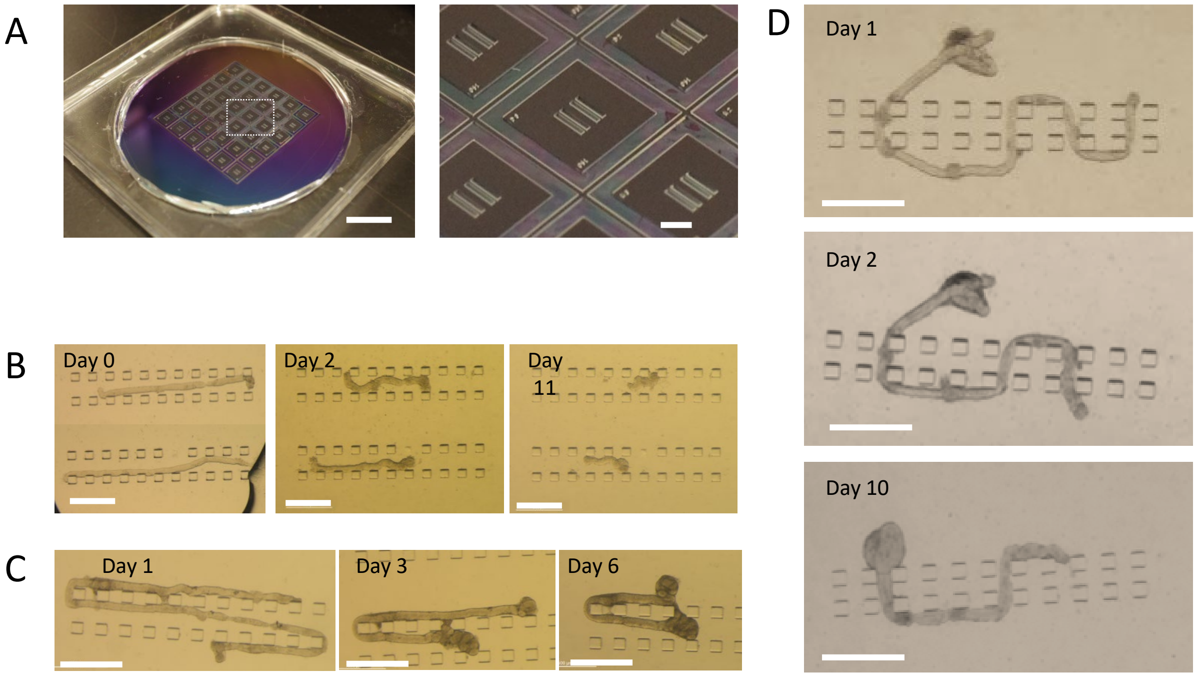

Seminiferous tubule PDMS ceiling (STPC) chip. (A) A mold for STPC chips. The dashed rectangular area is enlarged in the right panel, showing the mold for each single STPC chip. (B–D) Stereomicroscopic views of cut-isolated STs cultured beneath STPC chips under various settings. Scale bars: 2 cm (A left), 2 mm (A right), 500 µm (B–D).

S3 Fig. Schematic presentation of the protocol of the soft agarose method.

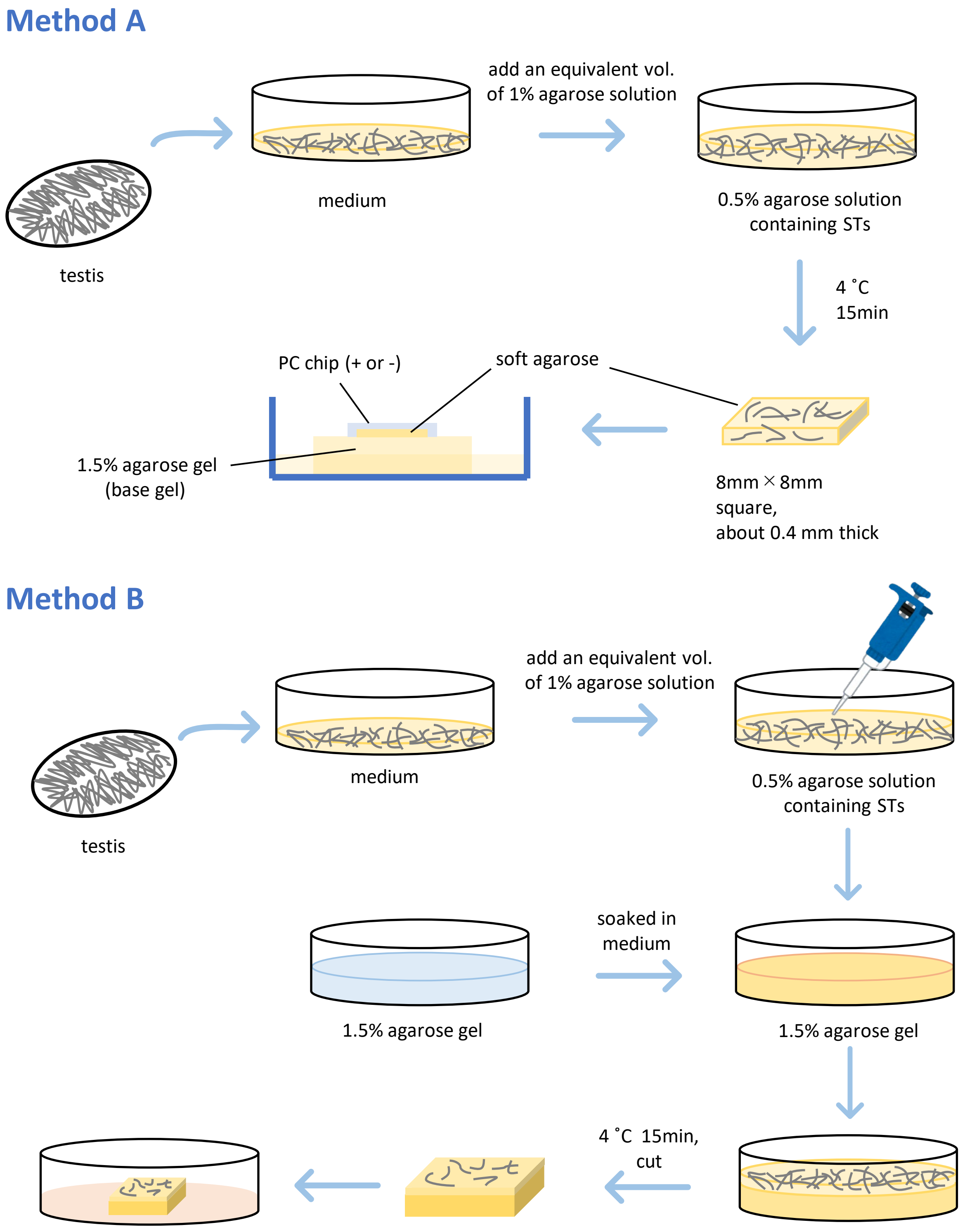

Schematic presentation of the protocol of the soft agarose method. In Method A, a soft agarose gel piece was cut out and transferred onto the base gel. In Method B, soft agarose was cooled down on the base gel. The two-layer gel was cut into squares and moved to a culture well. The volume of medium/agarose solution depended on the dish size.

S4 Fig. Effect of lower oxygen concentrations on spermatogenesis.

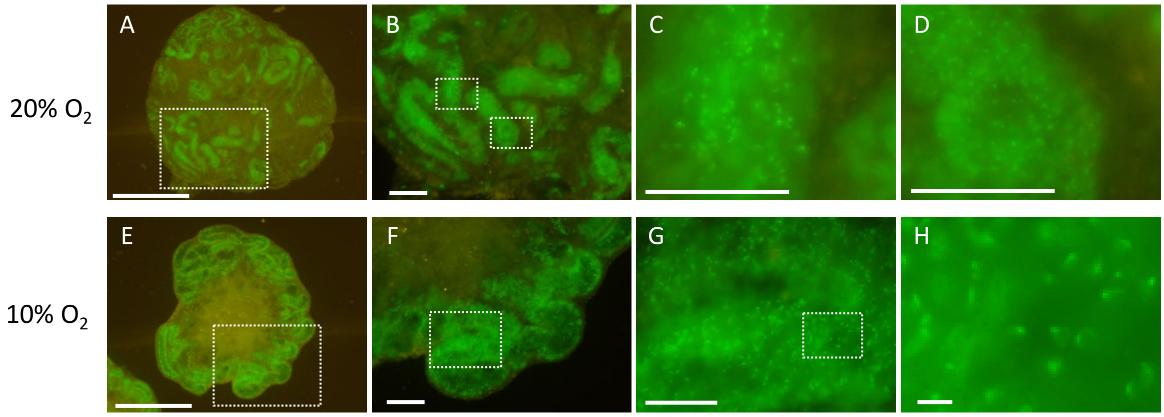

Effect of lower oxygen concentrations on spermatogenesis. Testis tissue masses of 1.5 dpp mice were cultured for 40 days at 20% or 10% O_2 concentration in an incubator. Dashed rectangular areas are enlarged in the adjacent right panels. Scale bars: 1 cm (A, E), 200 µm (B, F), 100 µm (C, D, G), and 10 µm (H).

S5 Fig. Effect of oxygen concentration on isolated STs and tissue masses.

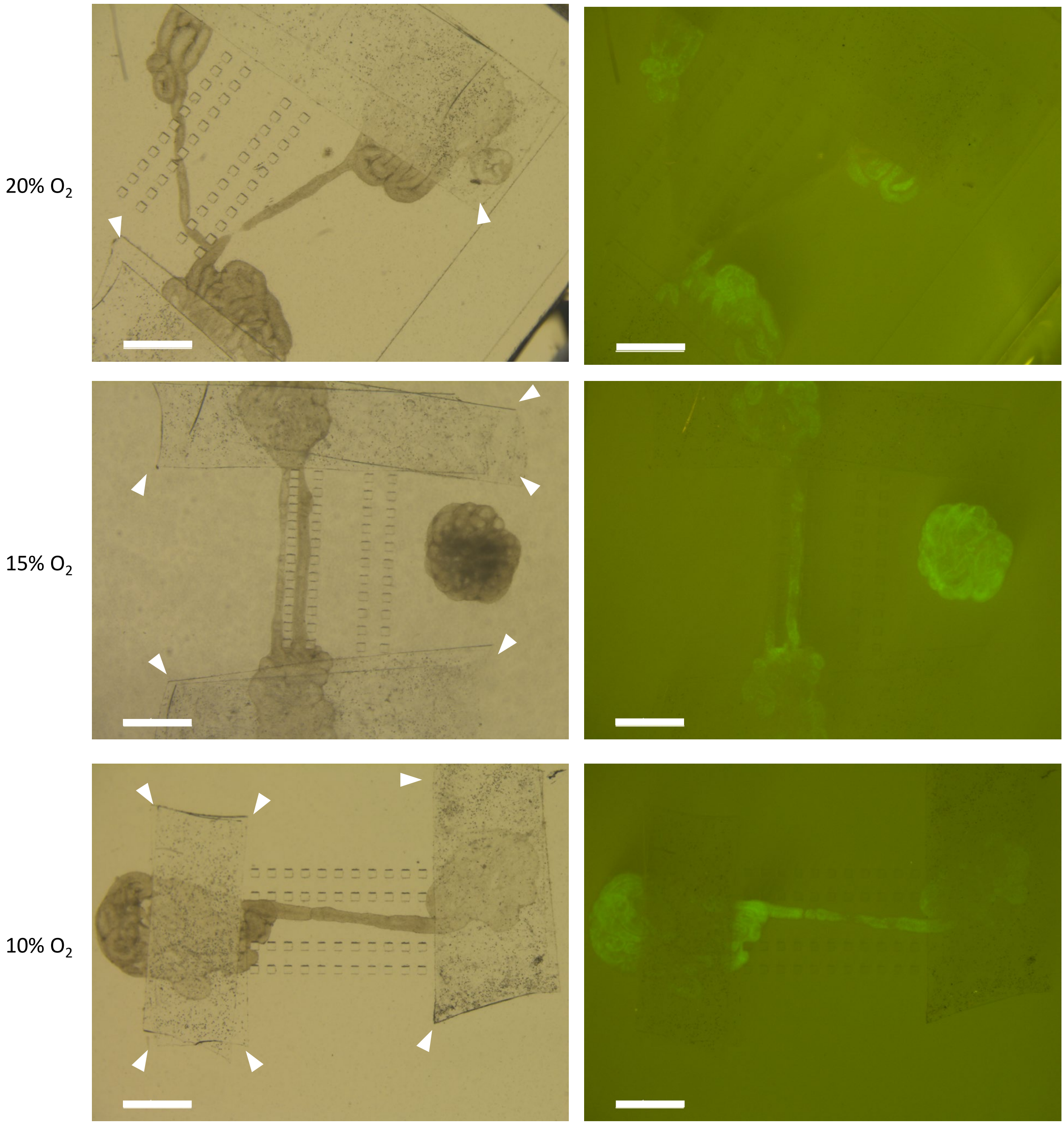

Effect of oxygen concentration on an isolated ST and tissue mass
Tissue masses showed a wide area of *Acr*-GFP expression, irrespective of O_2 concentration. The linearly isolated ST segments showed little GFP emission at 20% O_2, while those in 15% and 10% O_2 showed GFP. Polycarbonate porous membranes (arrowheads) were placed between the tissue mass and PDMS ceiling to hold the tissue in place. Scale bar: 1 mm.

S6 Fig. Culture methods summary.

		Acr-GFP positive rate (%) *					
		20% O_2		15% O_2		10% O_2	
PC	(1) Cut-isolated conglomerated ST	7 / 132	(5.3)				
	(2) Uncut-isolated ST (STPC chip)	16 / 36	(44.4)	31/41	(75.6)	25/27	(92.6)
		24 / 45	(53.3)				
	(3) Tissue mass culture	117 / 130	(90.0)				
Soft agarose	(4) Cut-isolated ST	72 / 268	(26.9)				
	(5) ST aggregate	55 / 90	(61.1)				

* Acr-GFP positivity of each sample was determined by whether or not the GFP-positive area comprised 10% or more of the total area.

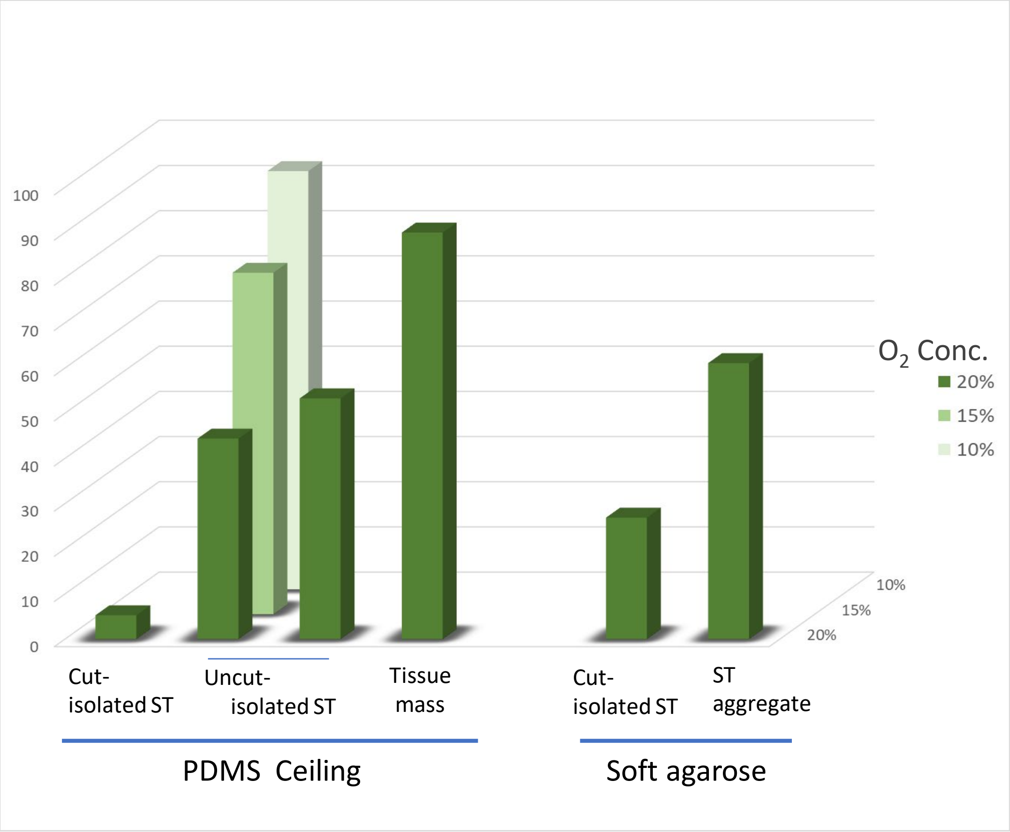

S1 Table. Antibodies used in this study.

Antibodies used in this study.

Antibody	Provider	Catalog number	Batch Number	Dilution
Rabbit anti-RFP polyclonal antibody	MBL	PM005	044	1:1000
Alexa Fluor 555 donkey anti-rabbit IgG	Thermo Fischer Scientific	A-31572	2088692	1:200
Anti-GFP rabbit polyclonal antibody conjugated with Alexa Fluor 488	Thermo Fischer Scientific	A-21311	2207528	1:50
Lectin PNA from Arachis hypogea (peanut), Alexa Fluor 647 conjugate	Thermo Fischer Scientific	L32460	1005954	1:400
Chicken anti-GFP antibody	Abcam	ab13970	GR3361051-13	1:1000
Mouse anti-SYCP3 antibody	Abcam	ab97672	GR191517-1	1:500
Goat anti-GFRα1 antibody	R&D Systems	AF560	BQE0518031	1:200
Rabbit anti-STRA8 antibody	Abcam	ab49602	GR32540003	1:250
Recombinant Anti-gamma H2AX antibody	Abcam	ab 81299	GR93354-25	1:500
Rabbit anti-mouse HSD3β polyclonal antibody	Trans Genic Inc.	KO 607	TG 060813	1:250
