# Supplementary material for: In vitro spermatogenesis in isolated seminiferous tubules of immature mice
Source: PLoS One. 2023 Apr 6;18(4):e0283773. doi: 10.1371/journal.pone.0283773 (PMC10079070; doi:10.1371/journal.pone.0283773)
Supplement: Attachment — Submitted filename: PONE-D-22-19509 comments.docx [file pone.0283773.s003.docx]

Comments:

Recapitulation of in vitro spermatogenesis is essential to study the molecular mechanisms and to address infertility. Feng et al. extended their previous studies on in vitro spermatogenesis using the ST culture method using Acr-GFP andH3 mice. The authors tried both minced or individual STs and aggregated STs. Interestingly, they found that aggregates or individual STs having aggregates at both ends show efficient spermatogenesis compared to individual STs. Further, using ST culturing, authors have shown the effect of oxygen concentration on spermatogenesis. This is a motivating extension work; however, some aspects of the study need to be clarified, and more experiments are necessary to describe the manuscript's findings. Also, serious issues with the writing must be addressed. Overall, the paper feels premature and would benefit from some major rewriting. Throughout the article, including the abstract, many vocabularies, grammar, and pose errors make it very hard to follow and interpret.

Major comments:

The abstract is poorly written.

If possible, I would suggest to the authors consider making the video of methods A and B. It will be beneficial to the researchers and readers.

Authors made major conclusions that spermatogenesis can be induced in short pieces of STs in culture. However, only 26% of the short pieces show the +ve signals of GFP and mcherry. Also, they are nearby small clustered regions. Of course, we can see the GFP signals but Mcherry and GFP colocalized signals we see nearby clusters only?

Here the point is with the low efficiency of spermatogenesis in short pieces, how come one can use this technique to study mechanisms of spermatogenesis or any toxicological studies?

Line 465: Numerous studies have described the pivotal roles of these cells in spermatogenesis. Leydig cells produce and secrete androgens and other factors, such as colony-stimulating factor 1 (CSF-1) [31]. The testicular macrophages also produce CSF-1 and enzymes involved in retinoic acid (RA) biosynthesis [32]. Lymphatic endothelial cells secrete fibroblast growth factors (FGFs), which maintain stem cell populations for spermatogenesis [33]. All these factors and others not yet identified could contribute to the promotion and maintenance of spermatogenesis and would be necessary for efficient spermatogenesis in vitro.

As the authors stated ST’s external microenvironment may play a critical role in spermatogenesis. Did the authors try to culture short pieces of STs with some of the factors like RA, CSF-1 or growth factors? In another way did the authors try to co-culture with Leydig cells or other cells required for spermatogenesis to improve the efficiency?

Minor comments:

Fig1G Y axis?

323 expression was observed in 55 out of 90 sa268mples (61.1%) (Fig 3D, E).

Fig 3G ; error bars labelled. Make it uniform or mention it in legends

Fig S1 and 2 methods 0.5% agarose solutions with STs kept in 4deg. How about cold stress or shock on tubules and spermatocytes?

Fig3: What % of aggregates were observed? What do the authors mean occasionally?

Line 327: In 40–55 days, STs from an experiment were examined by whole mount immunochemical staining. In 2 samples of cut-isolated ST, cells positive for both GFP and mCherry were observed out of 41 samples examined

The image shown +ve for GPF and Mcherry in cut isolate is not convincing. Because the +ve signals we see near the cluster.

Line 334: We then stained Leydig cells with anti-HSD3β antibody under whole mount condition. In fact, HSD3β-positive cells were detected abundantly in aggregated STs and only marginally in isolated ST samples (Fig 3I). Please add the quantification

Line 340: In our previous study using rat testis tissues, we found that O2 tension could influence the spermatogenic efficiency in vitro and O2 concentration lower than atmospheric 20% would be favourable : Rewrite

377 that this condition, namely uncut-isolated ST under 10%O2: 10% space

Overlapping scale bars

Make uniformity in scale bars

Fig2:

(B) Even though authors have mentioned that Acr-GFP expression in an ST showed unique oscillatory fluctuation, the authors need to explain the reason for sharp fluctuation in Acr-GFP expression from Day 24 to Day 31.

Fig3:

(F) The authors examined only 41 cut-isolated STs for both GFP –mCherry expression. What about the rest of the cut-isolated STs as the authors have found quite significant (72 nos) GFP expressing cut-isolated STs?

In lines 318, 319 and 320, they found small clumps of STs which they have considered aggregated STs. The authors need to clarify whether this is a human error or if these clumps are forming on their own. In the latter case, the authors also need to mention the frequency and percentage of finding these clumps.

In addition to this, the authors need to clarify whether they have included these clumped STs in their count for aggregated STs or not.

(I) The authors need to provide statistical analysis for this figure as they have stated that ‘aggregated STs may maintain interstitial cells between each ST, which could be a reason that aggregated STs supported spermatogenesis more favourably.’

Fig. 4

(B) Out of 20%, 15% and 10% concentrations, in 10% O2 GFP expression is more. Whether this is the optimal concentration for spermatogenesis or there is the possibility of more spermatogenesis below 10%? The author needs to clarify.
