# Supplementary material for: In vitro spermatogenesis in isolated seminiferous tubules of immature mice
Source: PLoS One. 2023 Apr 6;18(4):e0283773. doi: 10.1371/journal.pone.0283773 (PMC10079070; doi:10.1371/journal.pone.0283773)
Supplement: Attachment — Submitted filename: Reply to Reviewers.docx [file pone.0283773.s004.docx]

Reviewer #1: Comments:

Recapitulation of in vitro spermatogenesis is essential to study the molecular mechanisms and to address infertility. Feng et al. extended their previous studies on in vitro spermatogenesis using the ST culture method using Acr-GFP andH3 mice. The authors tried both minced or individual STs and aggregated STs. Interestingly, they found that aggregates or individual STs having aggregates at both ends show efficient spermatogenesis compared to individual STs. Further, using ST culturing, authors have shown the effect of oxygen concentration on spermatogenesis. This is a motivating extension work; however, some aspects of the study need to be clarified, and more experiments are necessary to describe the manuscript's findings. Also, serious issues with the writing must be addressed. Overall, the paper feels premature and would benefit from some major rewriting. Throughout the article, including the abstract, many vocabularies, grammar, and pose errors make it very hard to follow and interpret.

Major comments:

The abstract is poorly written.

Reply) I have rewritten the abstract to be more specific about what we did in this study. I think that the new abstract is more informative and easier to understand for most readers.

If possible, I would suggest to the authors consider making the video of methods A and B. It will be beneficial to the researchers and readers.

Reply) We have made a video showing the procedure of soft agarose methods A and B, which was attached as supplementary data. Thank you for your suggestion.

Authors made major conclusions that spermatogenesis can be induced in short pieces of STs in culture. However, only 26% of the short pieces show the +ve signals of GFP and mcherry. Also, they are nearby small clustered regions. Of course, we can see the GFP signals but Mcherry and GFP colocalized signals we see nearby clusters only?

Reply) Reading your comment, I came to notice that GFP- and mCherry-double positive cells were observed at locations in a tubule which was not stretched but bent or twisted to form ST aggregation-like configuration. This may have an important implication for a future study. In any case, however, they were present in each singly cut-isolated ST, but not in STs of clustered or aggregated.

Here the point is with the low efficiency of spermatogenesis in short pieces, how come one can use this technique to study mechanisms of spermatogenesis or any toxicological studies?

Reply) The Acr-GFP positivity rate of isolated STs is indeed lower than that of ST aggregates or tissue mass cultures. However, I consider this as follows. In order to improve culture condition for spermatogenesis, we have to test variety of interventions, including supplements in the medium and physical conditions like different O_2_ concentration. Currently, the regular testicular tissue culture method, namely tissue mass culture, has achieved an efficiency as high as 90% Acr-GFP positivity. With this system, it may be difficult to find new culture conditions that are better than the control condition. For example, even if the addition of substance X to the culture medium is effective in promoting spermatogonial proliferation and inducing spermatogenesis, the effect of substance X may be overlooked in experiments where there is little room for improvement, with Acr-GFP expression rates as high as 90% in the control group. On the contrary, in this context, low spermatogenic efficiency of the isolated ST culture method may be useful to find better culture conditions. In fact, effect of O_2_ concentration was examined vividly in the present study to show lower O_2_ is better than 20%.

Line 465: Numerous studies have described the pivotal roles of these cells in spermatogenesis. Leydig cells produce and secrete androgens and other factors, such as colony-stimulating factor 1 (CSF-1) [31]. The testicular macrophages also produce CSF-1 and enzymes involved in retinoic acid (RA) biosynthesis [32]. Lymphatic endothelial cells secrete fibroblast growth factors (FGFs), which maintain stem cell populations for spermatogenesis [33]. All these factors and others not yet identified could contribute to the promotion and maintenance of spermatogenesis and would be necessary for efficient spermatogenesis in vitro.

As the authors stated ST’s external microenvironment may play a critical role in spermatogenesis. Did the authors try to culture short pieces of STs with some of the factors like RA, CSF-1 or growth factors? In another way did the authors try to co-culture with Leydig cells or other cells required for spermatogenesis to improve the efficiency?

Reply) Experiments suggested here are all important trial in our next studies. We will certainly plan such studies with the isolated ST experiment. Another reviewer, Reviewer 2, suggested to read a very recent paper by Önen et al. which used transwell insert system to coculture bone marrow derived mesenchymal cells and the testis tissue. Such co-culture system can be used to test your suggestion.

Minor comments:

Fig1G Y axis?

Reply) The Y-axis represents the percentage of GFP-positive samples out of all samples cultured. The title of the Y-axis was added to Fig. 1G of the revised manuscript.

323 expression was observed in 55 out of 90 sa268mples (61.1%) (Fig 3D, E).

Reply) I’m sorry for this careless typing mistake. I have corrected it in the revised manuscript.

Fig 3G; error bars labelled. Make it uniform or mention it in legends

Reply) In revision, I have erased them to make it uniform in all figures.

Fig S1 and 2 methods 0.5% agarose solutions with STs kept in 4deg. How about cold stress or shock on tubules and spermatocytes?

Reply) In our experience, storage at 4°C for 15 minutes does not affect the culture results.

Fig3: What % of aggregates were observed? What do the authors mean occasionally?

Reply) Thank you for this comment. Now I think “occasionally” was an incorrect word to describe the case. It totally depends on how rigorously the practitioner disassembled seminiferous tubules from each other. It is possible to eliminate aggregates completely, but it takes time and labor. Thus, in practice, ST aggregates remained more or less in every case, to varying degrees.

Line 327: In 40–55 days, STs from an experiment were examined by whole mount immunochemical staining. In 2 samples of cut-isolated ST, cells positive for both GFP and mCherry were observed out of 41 samples examined

The image shown +ve for GPF and Mcherry in cut isolate is not convincing. Because the +ve signals we see near the cluster.

Reply) As I replied above, the sample was a singly cut-isolated ST in the soft agarose. The “cluster” you mentioned is supposed to be the result of ST being bent or twisted during the whole mount immunohistochemistry procedure.

Line 334: We then stained Leydig cells with anti-HSD3β antibody under whole mount condition. In fact, HSD3β-positive cells were detected abundantly in aggregated STs and only marginally in isolated ST samples (Fig 3I). Please add the quantification

Reply) I counted the number of HSD3β-positive cells in the picture. The data were described in the figure legend as follows.

“HSD3β signal was observed in cells adhered to the outside of the STs, either singly (arrows) or as clusters (arrow heads). In these pictures, such HSD3β-positive cells were counted to be 5 and 41 in cut-isolated and aggregated STs, respectively. In addition, in case of aggregated STs, a clumpy interstitial tissue containing about 20 HSD3β-positive cells was also observed (surrounded by braces).”

Line 340: In our previous study using rat testis tissues, we found that O2 tension could influence the spermatogenic efficiency in vitro and O2 concentration lower than atmospheric 20% would be favourable : Rewrite

Reply) I rewrote as below in the revised manuscript.

In our previous study using rat testis tissues, we found that an O_2_ concentration lower than atmospheric concentration of 20% induced spermatogenesis with higher efficiency [20].

377 that this condition, namely uncut-isolated ST under 10%O2: 10% space

Reply) Thank you for this comment. I placed a space at that position in the revised manuscript.

Overlapping scale bars

Make uniformity in scale bars

Reply) Scale bars were examined again and corrected. Thank you.

Fig2:

(B) Even though authors have mentioned that Acr-GFP expression in an ST showed unique oscillatory fluctuation, the authors need to explain the reason for the sharp fluctuation in Acr-GFP expression from Day 24 to Day 31.

Reply) Yes, it was surprising to see such a drastic change in GFP expression in a single ST in 7 days. I consider this phenomenon as follows. The Acr-GFP expression starts at stage Ⅳ pachytene spermatocyte. Therefore, STs on day 24 in the figure should have contained germ cells of stage Ⅳ spermatocyte and some more differentiated cells. However, those GFP-expressing germ cells were about to die and disappeared in next 4 days. On day 28, therefore, the ST became GFP-negative, containing only germ cells of stage Ⅲ spermatocyte and less differentiated germ cells. Over the next 3 days, the stage Ⅲ spermatocytes turned into stage Ⅳ spermatocyte and beyond, and on day 31, the ST became GFP-positive again.

Fig3:

(F) The authors examined only 41 cut-isolated STs for both GFP –mCherry expression. What about the rest of the cut-isolated STs as the authors have found quite significant (72 nos) GFP expressing cut-isolated STs?

Reply) We have not examined the rest 31 samples for the mCherry expression.

In lines 318, 319 and 320, they found small clumps of STs which they have considered aggregated STs. The authors need to clarify whether this is a human error or if these clumps are forming on their own. In the latter case, the authors also need to mention the frequency and percentage of finding these clumps.

Reply) Yes, the clump STs was named as “aggregated STs”. STs do not self-aggregate in the soft agarose. They were results of incomplete dissociation of STs by the practitioner.

In addition to this, the authors need to clarify whether they have included these clumped STs in their count for aggregated STs or not.

Reply) Yes, clumped STs were counted as aggregated ST.

(I) The authors need to provide statistical analysis for this figure as they have stated that ‘aggregated STs may maintain interstitial cells between each ST, which could be a reason that aggregated STs supported spermatogenesis more favourably.’

Reply) Aggregated STs are a kind of artifact, unintentionally generated in soft agarose experiments.

The characteristics of aggregated STs, i.e., size, density, number of stromal cells, etc., may vary widely among them. For this reason, we hesitated to examine them in any further detail, after whole mount immunohistochemistry was performed once. We merely proposed that a greater number of interstitial cells may be responsible for more efficient spermatogenesis in aggregated STs. Thus, this is not definitive nor the conclusion of this study. I made it clearer in the revised manuscript.

Fig. 4

(B) Out of 20%, 15% and 10% concentrations, in 10% O2 GFP expression is more. Whether this is the optimal concentration for spermatogenesis or there is the possibility of more spermatogenesis below 10%? The author needs to clarify.

Reply) I think it is possible that a particular O_2_ concentration which is lower than 10% could be optimal for spermatogenesis in single ST. From our present study, it seems a delicate issue and the optimal O_2_ concentration may change in each tissue depending on the tissue size (O_2_ consumption rate). I wrote this in the revised manuscript.

Reviewer #2: In this study the authors evaluated the effect of oxygen concentration on in vitro spermatogenesis in neonatal Acr-GFP/Histone H3.3.3-mCherry double (Acr/H3) transgenic mice testes, using tissue mass, ST aggregate and ST segment culture systems. Although it seems to be a have short follow up duration s (34 day long), the authors report that the lower oxygen tension is favorable for spermatogenesis and for elongating spermatid production, and they demonstrated a new ST segment culture method providing opportunity for elucidating regulatory mechanism of spermatogenesis. While these data are potentially of interest, the manuscript lacks principal points that require a major irevision in order to ensure the data are presented with more clarity and greater empirical support (including controls and detailed data).

Title:could be intentional in order to correctly orient the readers to the output of the study.

Reply) I interpret this comment to mean that the current title is ambiguous and does not include the conclusions of this study. So, I considered other titles.

Present title; “Mouse in vitro spermatogenesis in isolated seminiferous tubules”.

New title 1; “Mouse spermatogenesis can proceed in isolated seminiferous tubules”.

New title 2; “Spermatogenesis proceeded in isolated seminiferous tubules of immature mice”.

New title 3; “???”

Thinking again, I reached the original one would be the best. Yet, I’m open to suggestions.

Introduction: lacks current literature and it is written in a broad and unfocused manner with a book chapter style. Therefore the rationale (lack of knowledge in recent literature related to the research question) , the clear description of the problem and the hypothesis or the research question that will solve the problem should be clearly declared .

Reply) I rewrote the introduction which included recent literatures. As for the research question, we focus on a very simple question which is whether a singly isolated ST can induce and support spermatogenesis or not. The answer was turned out to be yes. To make the introduction to be compact, we omitted the description of double Tg mouse.

Materials and Method lacks the study design, the sample size with power analysis, the number of repeats taht is required in order to answer the research question objectively.

Reply) The Materials and Methods section was reorganized. In addition, to facilitate the understanding of the present study as a whole, I have made a table along with graph encompassing every method together and present it as S6 Fig.

Several concerns related to methodolody and the proff of concepts are as following.

1. Please provide the detailed information of PDMS ceiling chip which was used for seminiferous tubule culturing method (including size of pillars in WxLxH and distance between the pillars) in M&M section.

Reply) I added that information in the M&M section.

2. In M&M section, between lines 121-124, the usage of a thin porous polycarbonate membrane is stated in some cases for pressing testis tissues against the base agarose gel by placing them under the PDMS ceiling to hold the tissue in place. Please clarify the optimization of culture systems by explaining in which cases the membrane was used.

Reply) First of all, I made a mistake to have written that polycarbonate membrane was used to press tissue mass. It was not polycarbonate membrane but a PDMS membrane (75 μm thick, FS7075C, ASAHI RUBBER inc). So, it was corrected accordingly in the revised manuscript.

This procedure was not relevant to the optimization of culture condition. The membranes were placed on the tissue mass but not on the isolated part of the seminiferous tubule. Therefore, we do not think it affect the culture results, nor relating to the optimization of culture condition.

3. The all culture platforms used in study should be clarified in M&M section. Please change the title "Culture method" as "Culture methods" in line 136 and explain each culture methods as subtitles individually. The title of "Soft agarose method" in line 156 should also be under culture methods title as a subtitle.

Reply) I reorganized the M&M section. “Soft agarose method” was changed to “Culture methods - Soft agarose method” and a new section of “Culture methods – PDMS ceiling (PC) method” was added to explain the detail of uncut-isolated ST method.

4. In figures 2A and 2B, there is high background for GFP, that makes the labeling data very unreliable.

Reply) That is the best we can do now with our microscope and camera setting. Sorry.

5. The terminology for culture systems is not consistent. Each culture system should be named in M&M section and the same names should be used in the remaining parts of the manuscript.

Reply) I made their name consistent, which was summarized in S6 Fig.

6. In figure 4F, PAS staining micrograph is given only for uncut-isolated ST culture, it should be also given for the other culture platforms for the same culture duration. The oxygen concentration is also should be provided in figure legend.

Reply) In this study, we took Acr-GFP and H3.3-mCherry as the reliable parameter for evaluating spermatogenic progression. I think they are basically enough to support our conclusion. Immunohistochemical staining and histological PAS stain were also performed to confirm and reinforce the conclusion. That is the reason that PAS staining was only performed and shown in the final figure. The oxygen concentration 10% was provided in the figure legend of Fig. 4E & F.

7. It seems that there are four culture methods as (1) cut-isolated ST (PDMS ceiling), (2) uncut-isolated ST (PDMS ceiling with pillars), (3) ST aggregate (soft agarose), (4) tissue mass culture (air-liquid interphase). In results section, the comparison of these 4 methods should be provided (in figure 1G, 3E, 3H, 4B and 4H). In figure 1G, it should be stated which oxygen concentration is used and what is the culture time for that analysis.

Reply) I appreciate your attentive reading of our manuscript. Present our research may not be organized in an ordinary style, because I described not only the final form of our best method but also methods we tested in our endeavor to improve them. I thank you for giving me an opportunity to think it again for summarizing and comparing culture methods.

Now I summarized our method as follows, which was presented in a table in S6 Fig.

(1) cut-isolated conglomerated ST (PDMS ceiling)

(2) uncut-isolated ST (PDMS ceiling with pillars)

(3) tissue mass culture (PDMS ceiling)

(4) cut-isolated ST in soft agarose

(5) ST aggregate in soft agarose

As for Fig. 1G, the culture time for the analysis was variable indeed. Each experiment has different culture duration from 35 to 77 days. The Acr-GFP positive sign (>10% area) was taken whenever during the culture period. I have added this information in the figure legend.

The results lack the answers of the research questions and Discussion part itatement of limitations of the current study. The changes in metabolites (analysis of culture media by LC-MS etc.), the functionality of elongated spermatids in terms of fertilization (ROSI, ICSI) or genetic stability tests should be performed or may be added as limitations.

Reply) Yes, there are many open questions in this study. We started this study asking whether isolated ST can induce and support spermatogenesis or not. To this question, we answered yes, it is possible to culture isolated ST for inducing spermatogenesis, although with lowered efficiency than tissue mass culture. I added sentences about the limitation of this study in the Conclusion of revised manuscript.

In discussion section, there are missing references in terms of comparison of the efficiency of new cut-isolated ST and uncut-isolated ST culture systems. The following articles should be discussed in terms of in vitro spermatogenetic process:

- Önen S, Köse S, Yersal N, Korkusuz P. Mesenchymal stem cells promote spermatogonial stem/progenitor cell pool and spermatogenesis in neonatal mice in vitro. Sci Rep. 2022 Jul 7;12(1):11494. doi: 10.1038/s41598-022-15358-5. PMID: 35798781; PMCID: PMC9263145.

Reply) Thank you recommending this paper to read. It is really interesting that bone marrow derived mesenchymal stem cells cultured on the well-bottom have promotive effect on in vitro spermatogenesis of testis tissue placed above in the transwell insert. This may mean that same method can improve the efficiency of spermatogenesis in the ST culture experiment. I added a sentence with this reference in the discussion.

- Baert Y, Dvorakova-Hortova K, Margaryan H, Goossens E. Mouse in vitro spermatogenesis on alginate-based 3D bioprinted scaffolds. Biofabrication. 2019 Apr 26;11(3):035011. doi: 10.1088/1758-5090/ab1452. PMID: 30921781.

Reply) This paper is also interesting showing that testis cells once singly dispersed were reaggregated by bioprinting method, in which spermatogenic progression was observed in following cultivation. Thus, this study may indicate a future direction of our present study too.

- AbuMadighem A, Shuchat S, Lunenfeld E, Yossifon G, Huleihel M. Testis on a chip-a microfluidic three-dimensional culture system for the development of spermatogenesis in-vitro. Biofabrication. 2022 Apr 20;14(3). doi: 10.1088/1758-5090/ac6126. PMID: 35334473.

Reply) As above paper, this paper also showed culturing reaggregated testis cell mass in a microfluidic device. I included this and above paper together in the discussion.

Reviewer #3: In this study, the authors developed an optimized method to culture the segment of seminiferous tubule (ST), and they showed that ST culture could reach the elongating spermatids stage. The authors patiently troubleshot the strategies to culture ST. They carefully described the detailed processes of their trial and error to develop an optimized protocol. I found the study valuable because the authors describe how they overcame the technical difficulties. This study would become an important asset in the field. Readers can understand the key process of their optimization. Further, the study demonstrates the impact of oxygen concentration in culture and clarifies the benefit of a hypoxic condition.

1. Some sentences in the abstract sound awkward. Lines 27-19 "Theoretically,”, and the last sentence in Lines 32-33.

Reply) I appreciate your comments. I have rewritten the abstract and those sentences were removed. Thank you.

2. Supplementary figures can be organized in the order of descriptions in the result section. S2A can be S1.

Reply) The numbers were reordered in the revised version.

3. Each result section consists of a single paragraph. These sections can be reorganized into some paragraphs to improve their readability.

Reply) I do appreciate this suggestion. In the revision, the section before the last, Cultivation of cut-isolated STs using a soft agarose method, was divided into 3 paragraphs. The last section, Lowered oxygen concentration was favorable for spermatogenesis, was divided into 5 paragraphs.

4. In Fig.1F, it was shown that the frequencies of GFP-positive cells were variable, but these data are not clearly shown in Figure G. These two data can be better described to clarify the frequency of GFP-positive cells.

Reply) Thanks for this comment. To make the difference among three groups, I made two thresholds, 10% and 50% of GFP-positive area, under observation with the stereomicroscope. The graph in figure 1G was modified to show GFP-positive rates with both of these two thresholds.

5. Fig. S2: Please add labels: Acr-GFP and H3.3-mCherry.

Reply) The labels were changed accordingly. Thanks.

6. Line 316: mislabel “S1 Fig. Method B”.

Reply) Sorry for this careless mistake. It was corrected in the revised manuscript.

7. Line 334-336: This is just a comment. Leydig cells are the primary source of testosterone or androgens in males. Testosterone or androgens can be supplemented in future studies.

Reply) Yes, I think the supplementation of testosterone and other substances secreted by Leydig cells should be done in next experiment. Thank you.
