# Supplementary material for: In vitro spermatogenesis in isolated seminiferous tubules of immature mice
Source: PLoS One. 2023 Apr 6;18(4):e0283773. doi: 10.1371/journal.pone.0283773 (PMC10079070; doi:10.1371/journal.pone.0283773)
Supplement: Attachment — Submitted filename: Response to Reviewers.docx [file pone.0283773.s005.docx]

6. Review Comments to the Author

Please use the space provided to explain your answers to the questions above. You may also include additional comments for the author, including concerns about dual publication, research ethics, or publication ethics. (Please upload your review as an attachment if it exceeds 20,000 characters)

**Reviewer #1:** The authors have done a good job revising their manuscript, which is clearly improved and ready for acceptance. Overall, the authors addressed most of my previous comments.

Reply: I appreciate your kind comments and suggestions.

**Reviewer #2:** The revised manuscript has been improved to some extent, in terms of quality with reorganized Abstract, Introduction, M&M sections and terminology for culture methods. On the other hand, the reliability of data in terms of time points, oxygen concentrations and sample size are not clear. Following are the comments.

Reply: Thank you for your comments.

1) The authors might provide a more appropriate title for the manuscript including the culture method used for isolated seminiferous tubules that constitutes the main key word of the study. In the present title, “Mouse in vitro spermatogenesis in isolated seminiferous tubules”, the term of “isolated seminiferous tubules” might point the isolation of seminiferous tubules from an adult mouse. Also, the age of the mouse and effect of oxygen level should be indicated in the title. Here you can find some options as possible titles:

Reply: I appreciate your suggestion. I changed the title as follows:

“In vitro spermatogenesis in isolated seminiferous tubules of immature mice”

2) The authors are strictly recommended to provide a more reliable data for comparison of groups at same time points in figures 1G, 3E, 3H and 4B. For example, in figure 1G, if the culture time for the analysis is variable from 35 to 77 days, it is not possible to compare the efficacy of cut-isolated ST, uncut-isolated ST and tissue mass culture methods in terms of GFP expression since the progression of the spermatogenesis is changeable in in vitro conditions. The time point, sample size (n=?) and oxygen concentration must be given in legends of figures 1G, 3E, 3H and 4B.

Reply: According to your recommendation, I narrowed the range of dates for the analysis in Figure 1G. The examination range was limited to 10 days, from culture days 31 to 40, and some of the data changed a bit. As for Figure 3E, the dates of analysis were culture days 20 to 25, and this information was added to the figure legend. In the case of Figure 3H, the day of tissue fixation for immunohistochemistry was added to the figure legend. Finally, in Figure 4B, GFP-positivity was assessed over culture days 20 to 30, and mCherry expression was confirmed on day 30 or 36. This information was added to the figure legend. Sample sizes are shown in the graph and an appropriate explanation was added to the legends. The oxygen concentration is now given as 20% in the legends for Figures 1G, 3E, and 3H.

3) All of the comments should be answered one by one on the rebuttal letter; then should address to the revised manuscript's specified lines/paragraphs/pages where the question is clearly answered.

Reply: Thank you for the advice.

4) The language that is used both on the rebuttal letter and the manuscript definitely requires a major refinement and revision in terms of scientific approach, the medical terminology and grammar.

Reply: Yes, I wrote the rebuttal letter ourselves and perhaps should have sought editorial help. As for the manuscript, however, I sent it to a professional English-editing company, where it was proofread and corrected by a native-English speaking editor. I sent the current rebuttal letter along with the revised manuscript to the company again.

**Reviewer #3:** The authors fully addressed my previous comments. It became a solid study. One minor thing I found is that the abstract contains too much details now, I feel. It can be reorganized in a way readers can capture the big picture.

Reply: Thank you for your kind comments and suggestions. I revised the summary by removing some of the extraneous details. I hope you find that the revised version is more concise.
