# Supplementary material for: In vitro spermatogenesis in isolated seminiferous tubules of immature mice
Source: PLoS One. 2023 Apr 6;18(4):e0283773. doi: 10.1371/journal.pone.0283773 (PMC10079070; doi:10.1371/journal.pone.0283773)
Supplement: Attachment — Submitted filename: Response to Reviewers.docx [file pone.0283773.s006.docx]

PONE-D-22-19509R2

In vitro spermatogenesis in isolated seminiferous tubules of immature mice

PLOS ONE

6. Review Comments to the Author

Reviewer #2: It has been improved to some extent by reorganization of the title, figures and figure legends, and additional information of sample size and oxygen level. On the other hand, the reliability of data in terms of time points and sample size are still not clear.

1) In figure 4B, 20% (n= 36), 15% (n=41) and 10% (n=27) oxygen level is compared in terms of the GFP721 and mCherry positivity of seminiferous tubules. The big difference in sample size between the groups gives rise to thought about potential of bias, and it may cause some possible errors in statistical analyses.
Response: I appreciate your insightful comments, which prompted us to review the data and analysis in Figure 4B. The number of samples varied from group to group mainly due to the technical difficulty of preparing uncut isolated STs. Even though we prepared comparable numbers of samples in each group at the start of the experiment, some samples were lost prior to evaluation due to fragmentation or slipping of the ST apart from the frame of the PDMS pillars. Nonetheless, we considered that the number of samples and quantity of data were sufficient for a robust statistical analysis. In the process of reviewing the statistical analysis, we did find a careless mistake. In the data for mCherry, the p-value for the comparison of 20% versus 10% O_2_ should have been marked with a single asterisk (*) rather than a double asterisk (**). This was corrected. The exact P values were as follows.

2) In figure 4B, the authors stated that “GFP721 positivity was judged during culture days 20 to 30. The mCherry expression was confirmed on culture day 30 or 36.” Both 6 and 10-day difference in in vitro culture of seminiferous tubules is still excessive in order to collect a reliable data from the experiments for two reasons:
Response: The Acr-GFP expression was observed beginning as early as culture day 14, which corresponded to a mouse age of 17 dpp when a 3 dpp mouse testis was used, as in Fig. 2. We observed samples every 7 days and recorded the GFP positivity. By judging GFP over culture days 20 to 30, we were therefore provided two chances to observe GFP. If we had narrowed the period or selected one particular date for judgement, we could have misjudged samples as negative even though they expressed GFP on neighboring days. The data in Figure 2B show that such a risk was not merely hypothetical. That is, the ST in Fig. 2B showed GFP expression on days 24 and 31, but no GFP expression on day 28. As described in the manuscript, such fluctuation/oscillation in GFP expression can occur, and we regarded samples such as this as GFP-positive. This was our rationale for the choice of culture days 20 to 30 as judging period.

In the case of mCherry, magnification using an inverted microscope was required in order to faithfully detect the red fluorescence. Therefore, the time period for mCherry detection was at the end of each culture experiment, and we removed the tissue from the culture dish and placed it directly on a slide glass for observation. As mentioned above, in vitro spermatogenesis does not proceed as smoothly as that in vivo, and in our experience the appearance of mCherry is delayed for days or more. Thus, in the initial experiment, we chose culture day 30, which corresponds to a mouse age of 36 days, as the time point for judging mCherry positivity, because the testis-donor mouse was 6 days old. We observed one mCherry-positive ST in the 10% O_2_ group, and no mCherry-positive STs in the 15% and 20% groups. In later experiments using 5-day-old mice, we chose culture day 36, corresponding to a mouse age of 41 days, reasoning that that time point would be more appropriate for finding mCherry-positive spermatids. We found 4 mCherry-positive STs in the 10% O_2_ group and none in the other groups. In hindsight, therefore, culture day 36 was more appropriate for finding mCherry-positive spermatids than culture day 30. We agree with you that it would have been better to pinpoint a particular day for analysis when trying to detect mCherry, since we had to select a single day in any case. Nonetheless, culture days 30 and 36 were within the appropriate time frame for detecting mCherry fluorescence and yielded reliable data showing the superiority of 10% O_2_.

1- The duration of mouse spermatogenesis is approximately 34 days. In Acr-GFP/Histone H3.3.3-mCherry double (Acr/H3) transgenic mice, GFP expression starts from pachytene spermatocytes at stage 4 and mCherry is expressed in spermatids from step 11 onward. Since the content and the size of subgroups of germ cells changes within the 6 and 10-day time interval, it is not suitable to include the data collected from day 20 to 30, and 30 to 36.
Response: In our culture experiment, the progression of spermatogenesis was not as smooth as that in vivo. The first appearance of Acr-GFP should have been around culture day 15 when a 0–1 day neonate was used, but it could have been delayed several days. In addition, even if GFP fluorescence appeared once, it could have disappeared and then re-appeared as shown in Fig. 2B. Therefore, we thought we should take steps to ensure that we did not miss such GFP-positivity, even if was temporary. This was our rationale for choosing a 10-day time span as an observation period for judging GFP fluorescence.

For mCherry, as mentioned above, the determination can be made only once per experiment. Therefore, it was necessary to select one particular day for the analysis. The selection of this day is a difficult decision. If we had chosen a day too early for mCherry detection, i.e., a day when the most advanced germ cells in ST are round spermatozoa such as those in step 8, mCherry would not be detected in any sample at any oxygen concentration. On the other hand, if the day had been too late for mCherry detection, i.e., a day when step-11 spermatids had already appeared in the ST some time ago, the mCherry-positive spermatids would have degenerated and disappeared in the interim, and we would have lost the opportunity to detect them. In the present experiment, these concerns were realistic, because mCherry-positive spermatids appeared rarely and only in low numbers. In careful consideration of all the above, we chose days 30 and 36, which corresponded to mouse ages of 36 and 41 days, as the time points for observation. In both cases, we observed mCherry-positive STs, supporting the appropriateness of our choice of days for the analysis. Therefore, we think our choice was appropriate and the data showing the superiority of 10% O_2_ were reliable.

2-This time gap may affect the viability of germ cells, and number of labelled cells in the meantime.
Response: Time gaps of 6 or 10 days could indeed provide an opportunity for the germ cells to die and disappear, as you indicated. However, they would also provide a chance for reappearance. Such appearances and disappearances of germ cells at particular stages of differentiation were observed as fluctuations in GFP fluorescence in each ST during the culture experiment, and the pattern of these changes was specific to each ST. Therefore, it would be ideal to collect data for a long enough period, whether for GFP or mCherry detection, and then to integrate such data to make a proper determination. In the case of GFP expression, such an approach can be achieved by observing the cells with a stereomicroscope while maintaining the culture. Therefore, we chose 20 to 30 days as the period for GFP evaluation.

The authors are recommended to reperform the statistical analysis by using same time points and sample sizes in the groups.

Response: For the reasons described above, we chose 20 to 30 days as the period for the judgement of Acr-GFP. In the case of mCherry, culture days 30 and 36 were considered preferable for the reasons mentioned above. Finally, we should mention that we corrected a careless mistake found in Figure 4B (the p-value for the 20% versus 10% O_2_ comparison as for mCherry should have been marked with a single asterisk (*) rather than a double asterisk (**)). We appreciate your careful reading and kind comments on our manuscript.

Reviewer #3: (No Response)
