# Supplementary material for: In vitro spermatogenesis in isolated seminiferous tubules of immature mice
Source: PLoS One. 2023 Apr 6;18(4):e0283773. doi: 10.1371/journal.pone.0283773 (PMC10079070; doi:10.1371/journal.pone.0283773)
Supplement: Attachment — Submitted filename: Response to Reviewers 20230310.docx [file pone.0283773.s007.docx]

**Editor**

Thank you for submitting your manuscript to PLOS ONE. After careful consideration, we feel that it has merit but does not fully meet PLOS ONE’s publication criteria as it currently stands. Therefore, we invite you to submit a revised version of the manuscript that addresses the points raised during the review process.

Minor changes in the conclusion section shall be made as per the suggestion of the reviewer.

Response: According to the suggestions from Reviewer #2, we added a phrase, technical difficulty in handling STs, in the conclusion section as one of the limitations of our technique.

**Reviewer #2**

Comment 1: The authors provided a good explanation for concerns about the validity and reliability of the data due to differences in sample size and time points of the groups compared. On the other hand, those explanations should be clearly stated in the manuscript in order to increase the readability of the publication.

Response: Thank you for this important suggestion. We added the following passages (shown in red font) to the **Observations** section of the Materials and methods:

Cultured tissues and ST segments were observed at least once a week under a stereomicroscope equipped with an excitation light for GFP (LeicaM205 FA; Leica, Germany). *Acr*-GFP begins to be expressed in mice at around 15 dpp *in vivo*, but its expression could be delayed by several days *in vitro*. In addition, GFP emission can fluctuate at intervals of several days. Therefore, to avoid false-negative results, GFP positivity was not necessarily determined on a single day; in most cases, two observations with a 7-day interval were taken, resulting in an observation period of greater than 7 days. Samples showing GFP expression during those periods were considered GFP-positive. The GFP-positive portions in each stretch of ST were measured by visual approximation as 0%, 1–10%, 11–20%, 21–40%, 41–60%, 61–80% or 81–100%.

H3.3 mCherry appears beginning at around 28 dpp *in vivo*. *In vitro*, however, its expression is delayed days to weeks, depending on the sample. For the reliable identification of mCherry, each sample tissue was removed from the culture well, placed on a slide glass and observed with an inverted microscope (IX73; Olympus) or a confocal microscope (FV1000–MPE; Olympus). The observation timing was carefully determined in each case, taking the status of preceding GFP expression into account.

Comment 2: The authors corrected the error made in the statistical analysis and explained the reasons for changes in time interval and sample size used to evaluate the GFP and mCherry expression reliably and rationally. When the explanations are added to the limitation section, the article will be ready for publication in order to be clearly understood by the readers.

Response: In the conclusion, we added “technical difficulty in handling STs” as one of the limitations of this technique. In the Results section, we also added the following sentences to clarify the reason for the different numbers of samples among groups.

The number of samples varied among the three groups due to technical difficulties in the preparation of uncut-isolated STs. Namely, some samples were lost prior to evaluation due to fragmentation or slipping of the ST apart from the frame of the PDMS pillars. Nonetheless, this experiment clearly demonstrated that lower O_2_ concentrations were associated with a higher rate of GFP expression in a larger area.

We appreciate your comments. We believe the manuscript was substantially improved through your input.
